# Acute Rheumatic Fever: A Disease of the Past?

**DOI:** 10.1155/2020/1470697

**Published:** 2020-02-08

**Authors:** Robert L. Myette

**Affiliations:** Department of Pediatrics, Queen's University at Kingston Health Science Centre, Kingston, Ontario, Canada

## Abstract

*Introduction*. Acute rheumatic fever (ARF) is a manifestation of the nonsuppurative sequelae of *Streptococcus pyogenes* infection. Herein, two cases of ARF are presented to highlight that this disease is present in urban cities, can be diagnosed in otherwise healthy children, and that its diagnosis may be challenging, or marred with confounders, leading to delays in diagnosis. *Case Report*. Two unrelated children, age 7 and 9, presented to an urban hospital in Canada with unique manifestations of ARF. Diagnosis of ARF in the first patient was interrupted by a course of steroids which masked symptoms leading to therapeutic delays. The second patient presented with facial droop and symptoms thought to be viral, thus leading to misdiagnosis as Bell's palsy. *Discussion/Conclusion*. ARF is more common in underserviced and marginalized populations, which may lead clinicians in urban centers to overlook signs or symptoms suggestive of ARF because they no longer see this condition routinely, or they believe it is a disease of the past.

## 1. Introduction

*S. pyogenes* is a Gram-positive, non-spore-forming coccus organized in chains [1]. This bacterium is isolated from healthy humans and routinely colonizes the pharynx, rectum, and skin [1]. *S. pyogenes* can cause pharyngitis and requires treatment with antibiotics to prevent ARF, which for some is the natural progression of the illness [2]. Here, two patients are described that experienced delays in their diagnosis of ARF.

## 2. Case Presentation

Two unrelated children presented for care on separate occasions in 2018 with unique manifestations of ARF. The first child was an otherwise healthy, 9-year-old male, who on initial presentation had lower extremity pain, fatigue, and fever. His vital signs were otherwise normal. There was no rash, and no arthritic joints were noted. There was no heart murmur. His exam at the time revealed a well-nourished boy with no concerning findings besides the noted tenderness to his lower extremities. Influenza was suspected, and therefore he was discharged with antipyretic therapy. There was no blood work drawn at this time to assess for inflammatory markers, and no imaging was indicated. He was subsequently seen for an asthma exacerbation in a community emergency room a few days after this initial visit and was prescribed a 5-day course of prednisone. Following this, his asthma symptoms, and his previous “influenza-like” symptoms, resolved. The family then travelled on vacation during which he was noted to become fatigued with activity and his lower extremity pain returned. He was febrile during this time as well. When he did return to our care, two weeks had passed since his first presentation for lower extremity pain. At this point, he had frank polyarthralgia, fever, an erythrocyte sedimentation rate of 66 mm/hr, and a high-sensitivity CRP of 8.8 mg/dL. His ASOT was greater than 1600 IU/mL. His pharyngeal culture was positive for *S. pyogenes*. Echocardiography 10 days later revealed mitral valve involvement which, coupled with the elevated ASOT, positive throat culture, and minor criteria, afforded the diagnosis of ARF.

The second child, a 7-year-old female, was also previously healthy and presented with facial droop, drooling, and fever. Her parents reported there had been a preceding viral illness. Her examination was otherwise normal, aside from the previously mentioned signs. There were no arthritides, and no murmur was auscultated. She was diagnosed with Bell's palsy and sent home with supportive care. There was no blood work or imaging obtained at this visit. Unfortunately, she returned to care 1 week later with choreiform movements (noted to abate with sleep), emotional lability, and “Milkmaid's grip.” At this visit, her mother reported that her daughter previously had a nonpruritic, serpiginous rash approximately 2 months before the first encounter and this was preceded by an untreated pharyngitis. This was not elicited during the first encounter when she was initially diagnosed with Bell's palsy. Her pharyngeal culture was ultimately negative. She underwent MRI, which was reported as normal. She also underwent echocardiography which revealed mitral and tricuspid valve involvement. Her diagnosis of ARF was made in the setting of Sydenham chorea.

Both patients were seen by Paediatric Cardiology and Infectious Disease and were started on penicillin. They both have had repeat echocardiograms which showed improvement, but no resolution of their valvular disease.

## 3. Discussion

ARF is a manifestation of the nonsuppurative sequelae of *Streptococcus pyogenes* infection. The pathomechanism of ARF is most likely attributable to molecular mimicry, whereby the immune system attacks and damages body tissues [3]. In some urban centers, it may be regarded as a disease of the past, or one which only affects vulnerable and underserviced populations [4, 5]. Indeed, as it pertains to Canada, the incidence of ARF is 75 times higher in some Northern Ontario communities compared to the rest of the country [6]. This is largely related to overcrowding and poor access to care, leading to missed early diagnoses and appropriate intervention [6]. This is similar to rural and indigenous communities in Australia [5]. However, ARF is present in urban cities and if not considered and identified, may lead to delays in diagnosis and subsequent morbidity, or even mortality.

ARF can be diagnosed clinically using the Jones Criteria, originally made up of one set of major and one set of minor criteria. The major criteria included arthritis, carditis, erythema marginatum, subcutaneous nodules, and Sydenham chorea [7]. The original minor criteria included arthralgia (when the arthritis criterion is not met), prolonged PR interval (when the carditis criterion is not met), fever, or elevated inflammatory markers. Evidence of *Streptococcal* infection is paramount, unless the patient presents with Sydenham chorea. Important to note, two of the five major criteria, including erythema marginatum and subcutaneous nodules, are relatively uncommon. Arthritis is the most common presenting feature of ARF. Carditis is valvular in nature but may not manifest with murmur on clinical exam.

Despite these longstanding criteria for the diagnosis of ARF, there are still challenges. This was highlighted in a recent nationwide prospective review of ARF in Australia, where children deemed as non-high risk were being missed because of subtle or nonspecific presentations [5]. Because of issues like this, the original Jones Criteria were modified [8, 9], and they now stratify patients into low- and high-risk populations to allow for better diagnostic accuracy and to ensure patients at high risk of ARF who may not meet all original criteria are captured and investigated accordingly. Essentially, in low-risk populations, the major criteria are the same as the original Jones Criteria, namely, carditis (clinical or subclinical), arthritis (must be polyarthritis), chorea, erythema marginatum, and subcutaneous nodules [8]. In the low-risk population, minor criteria are as follows: polyarthralgia, hyperpyrexia (≥38.5°C), ESR ≥ 60 mm/hr and/or CRP ≥ 3.0 mg/dl, and prolonged PR interval (only if no carditis, age appropriate differences must be considered) [8]. The high-risk population criteria allow for better detection with less stringency. Major criteria include carditis (clinical or subclinical), arthritis (mono- or polyarthritis), polyarthralgia, chorea, erythema marginatum, and subcutaneous nodules. Minor criteria are similar to the low-risk minor criteria but have some differences. Specifically, the lower height of fever, at ≥38°C, monoarthralgia, ESR ≥30 mm/hr and/or CRP ≥3.0 mg/dl, and similar criteria for PR interval [8]. The reader is referred here [2, 9, 10] for other recent reviews.

Additional delays to diagnosis in the aforementioned study were related to ARF being mistaken for osteomyelitis or septic arthritis [5]. Indeed, arthritis is a nonspecific finding in ARF. Delays in diagnosis can also be related to poor access to care, intercurrent illness, or rare major criteria as primary manifestations [4, 11]. Rare major criteria such as subcutaneous nodules, if not observed, may result in prolongation of time to diagnosis in the setting of nonspecific arthritides and fever, as was seen in a recent case report [12]. This, however, is quite rare. Further, erythema marginatum may also be mistaken for more common rashes, or fungal infections such as *Tinea* [13]. Lastly, it is possible that exposure to medications such as antibiotics, analgesics, and antiinflammatory agents during the disease course may alter the classical presentation; however, this is not well covered in the literature.

A delay in diagnosis was seen in the first patient who received a 5-day course of prednisone with resolution of his ARF symptoms that had been initially misdiagnosed as influenza. He initially presented with some features consistent with ARF; however, because influenza is more commonly seen, ARF may not have been considered. Additionally, and in keeping with findings in the previously mentioned recent prospective study [14], this delay may also have been partly because the family did not believe the illness was severe. Treatment of an intercurrent asthma exacerbation with steroids then masked the ongoing symptoms of ARF that may have brought him back to care earlier, instead of 2 weeks later. Therefore, it is important to consider ARF even in normally serviced communities and test if there is clinical suspicion. Similarly, delays in diagnosis for the second patient were related to the initial presentation of facial droop, which is a rare, but described, manifestation of ARF [15]. This delay was further compounded by the family not seeking care because they thought her illness was “a routine cold.” Given the inability to capture all biochemical and imaging markers of ARF at the initial clinic visits, it is possible that the initial presentations were not consistent with ARF and these prediagnosis symptoms were those of a separate illness prior to the development of ARF. However, temporally, it would appear that they were early signs of ARF that were missed given the relative rarity of this condition at some urban centers.

An important aspect to also consider is the fact that these patients were living in an urban city and did not have any classic risk factors for ARF that are present in Northern communities, or those populations that are underserviced. Additionally, they were not related or going to the same school. As such, it is always important to consider ARF in the right clinical context, despite its usual characterization as a disease of the past in normally serviced populations. It is true that these patients had unique presentations which may have contributed to their delays in diagnosis; however, major pieces of history were not elicited and important biochemical tests were not performed at the first encounter which may have allowed for an earlier diagnosis. It is impossible to say whether these patients would have been identified and treated earlier if they came from a community where ARF is more prevalent, or if the high-risk population criteria had been applied.

## 4. Conclusion

Acute rheumatic fever is not a disease of the past and is not limited to populations that are underserviced or marginalized. Its presentation may be insidious or altered by concomitant medications or disease processes. This report can serve as a reminder that rarity is contextual, and diseases of the past are evermore diseases of today.
